# High-Flow Nasal Cannula (HFNC) Versus Conventional Oxygen Therapy in the Prevention of Post-extubation Hypoxemia: A Systematic Review and Network Meta-Analysis

**DOI:** 10.7759/cureus.104096

**Published:** 2026-02-23

**Authors:** Khaled A Soliman, Rafie Ahmed, Abdulmohsen Alkhalagi, Souhail Mansouri, Faris H Radman, Ali H Alharbi, Mohamed Hefny, Abeer Hareeqah, Haitham A Althawab, Saeed M Oraydah, Nehal O Alanzi, Shahad F Al-Smah, Ali S Metwaly

**Affiliations:** 1 Emergency Medicine, Armed Forces Hospital Southern Region, Khamis Mushait, SAU; 2 Nursing, College of Applied Medical Sciences, Taiz University, Taiz, YEM; 3 Respiratory Medicine, King Abdullah Medical Complex, Jeddah, SAU; 4 General Practice, Al Noor Specialist Hospital, Makkah, SAU; 5 Respiratory Medicine, Taibah University, Madinah, SAU; 6 Internal Medicine, Al Hussein University Hospital, Al-Azhar University, Cairo, EGY; 7 Pharmacy, Madinah Health Cluster, Madinah, SAU; 8 Emergency Medicine, Qatif Central Hospital, Qatif, SAU; 9 General Practice, Medical Services: Ministry of Interior, Riyadh, SAU; 10 Emergency Nursing, Saudi Arabia Ministry of Health, Arar, SAU; 11 General Practice, Najran University, Najran, SAU; 12 Medicinal Chemistry and Drug Discovery, Faculty of Pharmacy, Alexandria University, Alexandria, EGY

**Keywords:** conventional oxygen therapy, critically ill patients, high-flow nasal cannula, network meta-analysis, noninvasive ventilation, post-extubation, reintubation, weaning

## Abstract

Respiratory failure following extubation is a major contributor to patient morbidity and death in intensive care settings. While various methods exist, the relative effectiveness of high-flow nasal cannula (HFNC), noninvasive ventilation (NIV), and conventional oxygen therapy (COT) in preventing reintubation remains an area of active research. This systematic review and network meta-analysis assessed these techniques in adult patients after scheduled extubation. A search of MEDLINE, Embase, and Cochrane CENTRAL was performed for relevant randomized controlled trials (RCTs) available up to December 2025. Data extraction was performed independently by two researchers. To synthesize findings, a random-effects network meta-analysis was employed, focusing on reintubation rates within a 72-hour window as the primary endpoint. Results were expressed as risk ratios (RR) accompanied by 95% confidence intervals (CI), and the Confidence in Network Meta-Analysis (CINeMA) tool was used to evaluate evidence quality. The analysis included 14 RCTs involving a total of 4,146 participants. Results indicated that both NIV (RR 0.56; 95% CI 0.34-0.94) and HFNC (RR 0.68; 95% CI 0.47-0.97) significantly decreased the reintubation risk when compared to COT. No significant difference in efficacy was observed between HFNC and NIV (RR 1.20; 95% CI 0.76-1.89). Based on surface under the cumulative ranking curve (SUCRA) rankings, NIV emerged as the most effective intervention (88% probability), followed by HFNC (60%) and then COT (2%). The certainty of evidence was moderate for comparisons involving COT, but low for the HFNC versus NIV comparison because of imprecision. HFNC and NIV are more effective than COT at preventing reintubation in the ICU. HFNC serves as a dependable preventative measure due to its ease of application and performance comparable to NIV. These findings support a shift away from standard COT toward more advanced noninvasive respiratory support.

## Introduction and background

Discontinuing invasive mechanical ventilation is a critical juncture in ICU care. While most patients successfully return to spontaneous breathing, a significant subset experiences post-extubation respiratory distress, with approximately 10% to 20% requiring reintubation [1,2]. This failure is often driven by a sudden loss of positive intrathoracic pressure upon endotracheal tube removal, leading to a decrease in functional residual capacity (FRC), alveolar derecruitment, and atelectasis [3]. Furthermore, post-extubation pathophysiology is compounded by diaphragm dysfunction, impaired mucociliary clearance, and upper airway edema, all of which precipitate a vicious cycle of increased work of breathing and worsening hypoxemia [3,4]. The clinical and economic burdens of postoperative pulmonary complications (PPCs) are substantial, with reintubation independently associated with increased hospital length of stay, higher healthcare costs, and elevated mortality [5].

Prophylaxis involves conventional oxygen therapy (COT) via Venturi masks or standard nasal prongs, especially for those with chronic obstructive pulmonary disease (COPD). However, the utility of these devices is restricted by design flaws that can impede patient stability, as the delivery of cold, dry gas by low-flow systems can cause patient discomfort and disrupt mucociliary function [6]. Moreover, COT flow rates frequently fall short of the patient’s peak inspiratory demand; this mismatch causes room air entrainment, leading to an unpredictable and fluctuating fraction of inspired oxygen (FiO2) [6,7]. As a result, COT is unable to offset physiological dead space or generate positive airway pressure, leaving fragile patients at risk for respiratory fatigue and hypoxemia [7].

As a potent alternative to standard oxygenation methods, clinicians are increasingly turning to the high-flow nasal cannula (HFNC). By providing heated and humidified oxygen at high flows (reaching 60 L/min), this modality matches or exceeds inspiratory demands, guaranteeing a consistent and precise FiO2 [7]. Compared to standard therapy, HFNC provides unique physiological advantages. It generates a flow-dependent positive end-expiratory pressure (PEEP) that helps maintain alveolar patency and prevent atelectasis. Furthermore, the continuous high-velocity airflow improves ventilation efficiency by washing out carbon dioxide from the nasopharyngeal dead space [8]. The superior humidification offered by HFNC also protects mucociliary transport and improves comfort relative to the dry gas of COT or the restrictive interfaces used in noninvasive ventilation (NIV) [7,8]. Although NIV effectively averts respiratory failure in certain high-risk groups, its use is constrained by claustrophobia, intolerance, and complications such as skin breakdown or gastric distention [4,8].

Despite the theoretical benefits of HFNC, clinical evidence regarding its efficacy compared to that of COT remains heterogeneous. Previous meta-analyses have suggested potential benefits in reducing reintubation rates and improving oxygenation; however, the results have varied across surgical and medical populations [1]. Furthermore, the relative efficacy of HFNC compared to NIV remains a subject of debate, particularly in defining the optimal hierarchy of noninvasive respiratory support strategies for preventing post-extubation hypoxemia [2,9]. To address these uncertainties, a systematic review and network meta-analysis (NMA) were conducted. This approach allows for the simultaneous comparison of HFNC, COT, and NIV, integrating direct and indirect evidence to provide a robust synthesis of the most effective strategies for managing patients during the critical post-extubation period.

## Review

Methods

Protocol and Registration

This NMA and systematic review was conducted in accordance with the Preferred Reporting Items for Systematic Reviews and Meta-Analyses (PRISMA) guidelines specifically designed for network meta-analyses [10]. The study protocol was formally registered in advance with the PROSPERO database (CRD420261282845) [11].

Search Strategy and Selection Criteria

A search strategy was executed across major databases to identify randomized controlled trials (RCTs) comparing HFNC with COT or NIV. Studies involving adult patients (>18 years) who underwent extubation from invasive mechanical ventilation were included. The primary outcomes of interest were post-extubation hypoxemia and reintubation rates. Studies involving pediatric patients, unplanned extubations, patients with tracheostomies, and observational studies were excluded. Study selection involved two independent authors screening titles, abstracts, and full manuscripts. Agreement between reviewers was assessed using Cohen’s kappa coefficient (κ) to verify consistency [12].

Risk of Bias Assessment

The quality of the included RCTs was evaluated using the Cochrane Risk of Bias (RoB 2) instrument [13]. This tool assesses five areas: the randomization procedure, deviations from planned protocols, missing data, outcome measurement, and reporting bias. Trials were then classified as having "low risk", "some concerns", or "high risk" regarding their overall bias.

Statistical Analysis

All analyses were performed using R software (version 4.5.1) [14]. To account for methodological variations, a random-effects model was applied for both pairwise and network comparisons. The Hartung-Knapp-Sidik-Jonkman (HKSJ) adjustment was utilized to refine variance estimation and minimize Type I errors, particularly given the limited number of studies [15,16]. Dichotomous outcomes, such as reintubation, were calculated as risk ratios (RR), while continuous variables (e.g., ICU length of stay) were analyzed using mean differences (MD). All results include 95% confidence intervals (CI). Also, 95% prediction intervals were calculated to estimate the range of true effects in future studies [17].

Heterogeneity and Inconsistency

Statistical heterogeneity within direct pairwise comparisons was quantified using the I2 statistic and between-study variance (τ2) [18]. The transitivity assumption was assessed by qualitatively comparing the distribution of potential effect modifiers (e.g., severity of illness and duration of mechanical ventilation) across comparisons [19]. The inconsistency between direct and indirect evidence within the network was evaluated using the node-splitting method, which separates evidence from direct comparisons and indirect sources to test for statistical disagreement [20].

Network Meta-Analysis and Ranking

A frequentist NMA was conducted to compare the relative efficacies of HFNC, COT, and NIV. The treatment hierarchy was determined by calculating the surface under the cumulative ranking curve (SUCRA). A SUCRA score approaching 100% suggests a higher probability that an intervention is the superior treatment option [21].

Assessment of Reporting Bias and Small-Study Effects

Reporting and dissemination biases were evaluated using comparison-adjusted funnel plots to detect asymmetry indicative of small study effects or publication bias specific to the network structure [22].

Additional Analyses and Robustness

Sensitivity analyses were performed by excluding studies with a high risk of bias to ensure the robustness of the findings. Subgroup analyses and meta-regression were conducted to explore the influence of potential moderators, such as surgical versus medical admission status and baseline hypoxemia severity. Furthermore, to assess whether the accumulated evidence was sufficient to reach a definitive conclusion and to control for random error associated with repetitive testing, a trial sequential analysis (TSA) was performed to calculate the required information size [23].

Certainty of Evidence

The Confidence in Network Meta-Analysis (CINeMA) tool [24] was used to assess the certainty of our findings. This system is an adaptation of the GRADE (grading of recommendations assessment, development, and evaluation) methodology [25] for network analysis and evaluates six key areas: bias within studies, dissemination bias, indirectness, lack of precision, heterogeneity, and network incoherence.

Results

Study Selection and Characteristics

**Figure 1:**
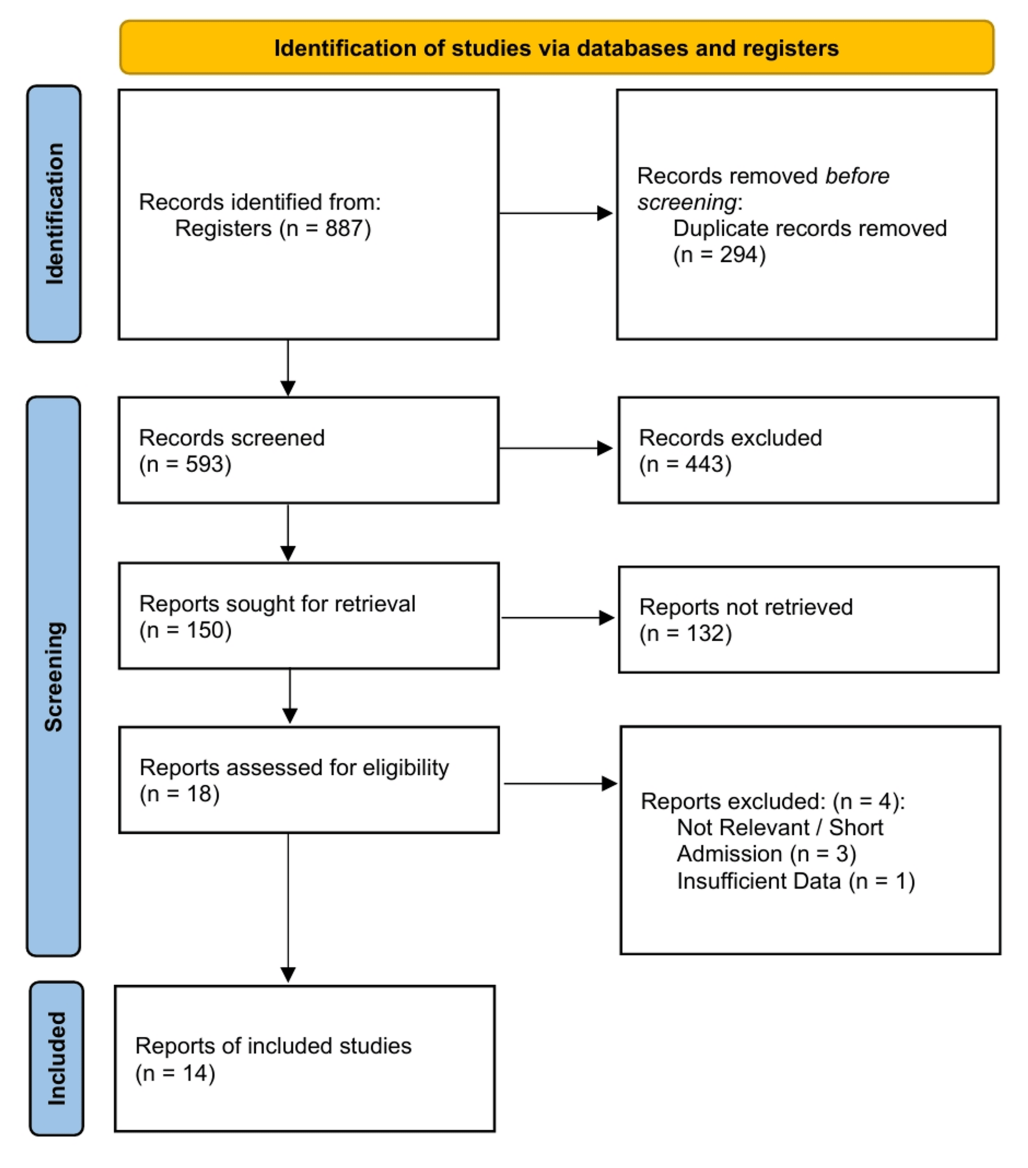
PRISMA 2020 flow diagram Abbreviation: PRISMA, Preferred Reporting Items for Systematic Reviews and Meta-Analyses

The systematic search and selection process yielded 14 RCTs eligible for inclusion in the network meta-analysis, comprising a total of 4,146 unique patients (Figure 1) [26-39]. The network geometry formed a closed loop involving three interventions: HFNC, COT, and NIV. Direct comparisons were available for HFNC vs. COT (9 studies [26-30,32,34,35,37]), HFNC vs. NIV (2 studies [31,33]), and NIV vs. COT (2 studies [38,39]). One study employed a crossover design and was included for physiological outcomes but excluded from reintubation analysis due to zero events in both arms [36]. Most studies recruited patients with mixed or high-risk profiles for extubation failure, including those with hypercapnia, obesity, or prolonged mechanical ventilation (Table 1).

**Table 1:** Baseline characteristics and study design of included randomized controlled trials (n=14) Abbreviations: HFNC, High-Flow Nasal Cannula; COT, Conventional Oxygen Therapy; NIV, Noninvasive Ventilation; PaO2/FiO2, ratio of arterial oxygen partial pressure to fractional inspired oxygen.

Study ID	Country	Population / Setting	Sample Size (N)	Interventions (Arm 1 vs. Arm 2)	Protocol Definition (HFNC)	Primary Outcome
Shaalan et al. [26]	Egypt	Surgical (Abdominal)	146	HFNC vs. COT	30–60 L/min, 37°C	Reintubation (72h)
Maggiore et al. [27]	Italy/France/ Spain/Greece	Mixed (Hypoxemic)	494	HFNC vs. COT (Venturi)	50–60 L/min, 37°C	Reintubation (72h)
Fernandez et al. [28]	Spain	Mixed (High Risk)	155	HFNC vs. COT	Started 40 L/min	Respiratory Failure (72h)
Song et al. [29]	China	Medical (Acute Resp. Failure)	60	HFNC vs. COT	Started 60 L/min	Success of Oxygen Therapy
Hernández et al. [30]	Spain	Mixed (Low Risk)	527	HFNC vs. COT	Started 10 L/min, titrated up	Reintubation (72h)
Hernández et al. [31]	Spain	Mixed (High Risk)	604	HFNC vs. NIV	Started 10 L/min, titrated up	Reintubation (72h)
Futier et al. [32]	France	Surgical (Abdominal)	220	HFNC vs. COT	50–60 L/min	Hypoxemia (1h)
Stéphan et al. [33]	France	Surgical (Cardiothoracic)	830	HFNC vs. NIV	50 L/min, FiO2 50%	Treatment Failure
Corley et al. [34]	Australia	Surgical (Cardiac, Obese)	155	HFNC vs. COT	Up to 50 L/min	Atelectasis Score
Maggiore et al. [35]	Italy	Mixed (Hypoxemic)	105	HFNC vs. COT (Venturi)	50 L/min	PaO2/FiO2 ratio
Rittayamai et al. [36]	Thailand	Medical (Crossover)	17	HFNC vs. COT	35 L/min	Dyspnea / Physiologic
Parke et al. [37]	New Zealand	Surgical (Cardiac)	340	HFNC vs. COT	45 L/min	PaO2/FiO2 ratio
Ferrer et al. [38]	Spain	Medical (Hypercapnic)	106	NIV vs. COT	N/A	Respiratory Failure
Nava et al. [39]	Italy	Mixed (High Risk)	97	NIV vs. COT	N/A	Reintubation

Risk of Bias Assessment

**Figure 2:**
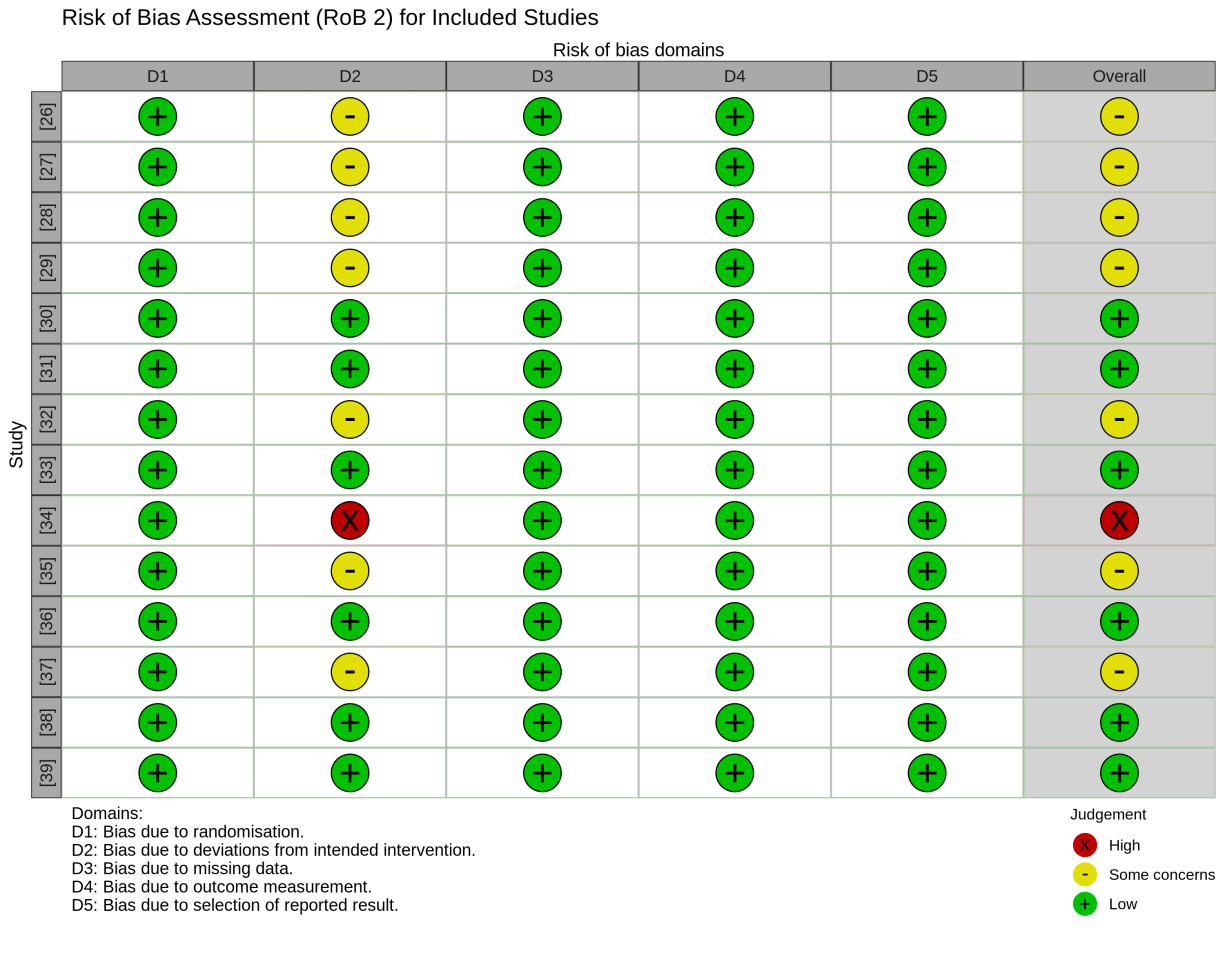
Risk of bias assessment (RoB 2) Traffic light plot illustrates domain-level quality assessments for the included RCTs [26-39] using the Cochrane RoB 2 tool (Sterne et al.) [13]. Green, yellow, and red symbols correspond to low risk, some concerns, and high risk of bias, respectively.

**Figure 3:**
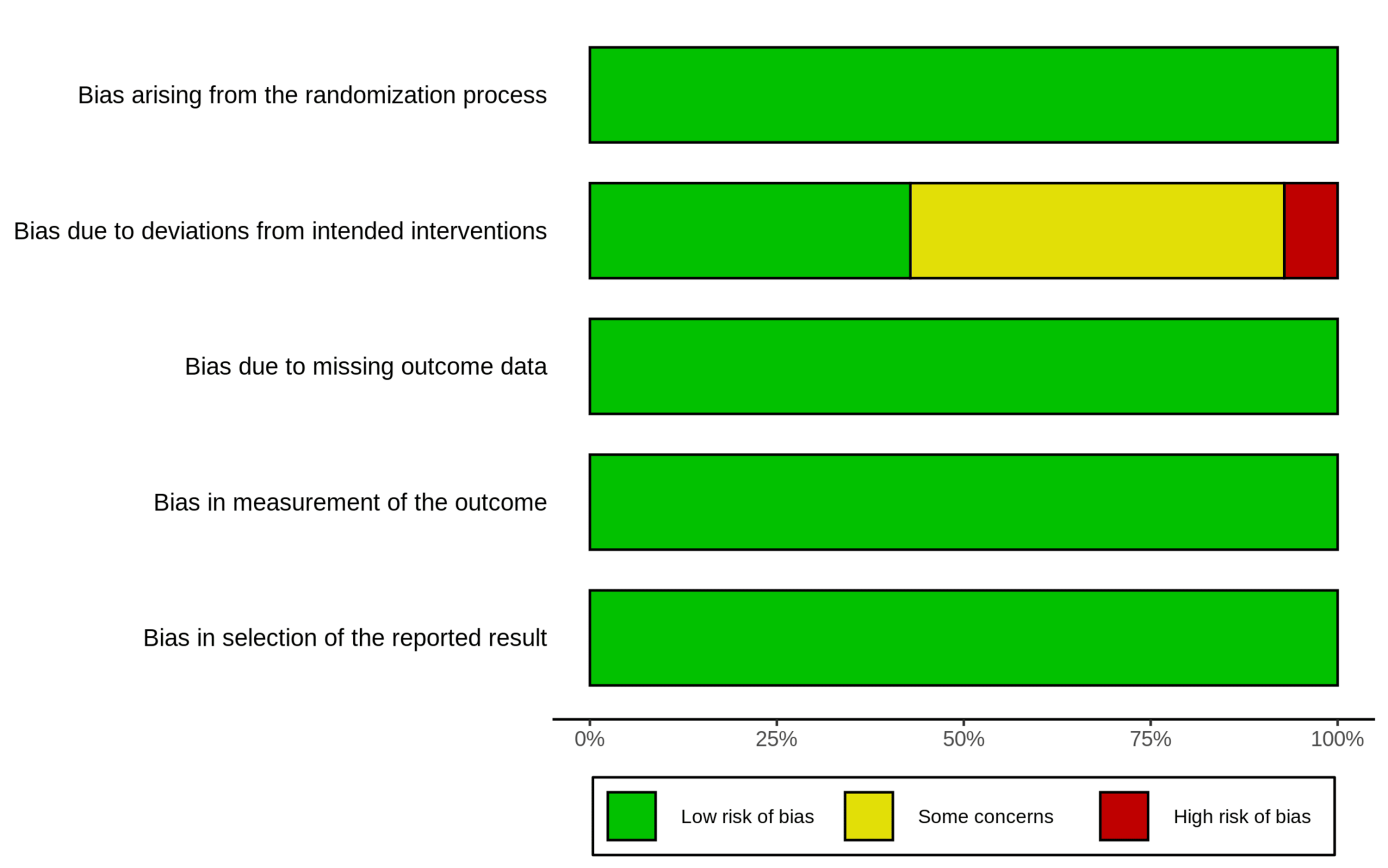
Weighted risk of bias summary plot of the overall risk of bias across all included studies

Methodological quality was evaluated using the Cochrane RoB 2 tool (Figure 2). Ten of the included trials were classified as having either some concerns or a high risk of bias. This rating was influenced by the domain regarding deviations from intended interventions (Figure 3). This issue is intrinsic to trials involving respiratory support devices, as blinding the interface (nasal cannula versus face mask) is impossible for clinical staff and patients. However, most studies mitigated detection bias by employing objective, pre-defined criteria for reintubation.

Primary Outcome: Reintubation Rate

**Figure 4:**
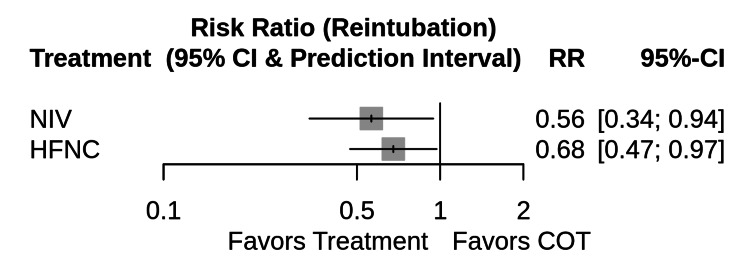
Network meta-analysis forest plot for reintubation Forest plot displaying the risk ratios (RR) and 95% confidence intervals (CI) for reintubation. The results are derived from a random-effects network meta-analysis using conventional oxygen therapy (COT) as the reference comparator. An RR of <1 favors the intervention over COT. Abbreviations: HFNC, High-Flow Nasal Cannula; NIV, Noninvasive Ventilation; COT, Conventional Oxygen Therapy.

The network meta-analysis demonstrated significant differences in efficacy among the interventions (Figure 4). Analysis revealed that HFNC provided a statistically significant reduction in reintubation rates relative to COT (Network RR 0.68; 95% CI, 0.47-0.97). Similarly, NIV demonstrated superior efficacy over COT (Network RR, 0.56; 95% CI, 0.34-0.94). There was no statistically significant difference in reintubation rates between the HFNC and NIV groups (Network RR 1.20; 95% CI, 0.76-1.89), suggesting comparable efficacy between these two noninvasive modalities.

The prediction interval for the HFNC vs. COT comparison ranged from 0.29 to 1.58, indicating that while the average effect is beneficial, heterogeneity in future populations (e.g., varying risk profiles) could result in non-significant findings in specific settings.

Treatment Ranking (SUCRA)

The SUCRA analysis indicated that NIV had the highest probability of being the most effective treatment for preventing reintubation (P-score = 0.88), followed by HFNC (P-score = 0.60). COT was ranked as the least effective treatment (P-score = 0.02).

Assessment of Transitivity and Heterogeneity

**Figure 5:**
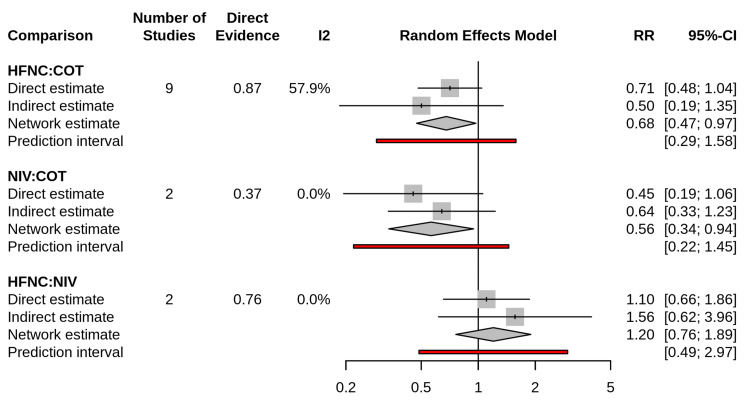
Node-splitting analysis for consistency Forest plot illustrating the agreement between direct evidence (derived from head-to-head randomized controlled trials) and indirect evidence (derived from network loops) for each pairwise comparison. The gray squares represent the effect estimates (risk ratio) with 95% confidence intervals for direct and indirect comparisons. The diamonds represent the network meta-analysis summary estimate. No statistically significant inconsistency was observed for any comparison (p>0.05 for all tests of disagreement), supporting the validity of the network transitivity assumption. Abbreviations: HFNC, High-Flow Nasal Cannula; NIV, Noninvasive Ventilation; COT, Conventional Oxygen Therapy; RR, Risk Ratio; CI, Confidence Interval.

Transitivity assessment revealed a balanced distribution of effect modifiers across network comparisons. The mean age of participants ranged from 47.4 to 68.5 years, and the proportion of male participants was comparable across direct comparisons (HFNC vs. COT: 63.8%; HFNC vs. NIV: 65.2%). However, moderate statistical heterogeneity was observed, particularly in the direct comparison between HFNC and COT (I^2^=57.9%), while other comparisons showed negligible heterogeneity (I2=0.0%), as detailed in Figure 5.

**Figure 6:**
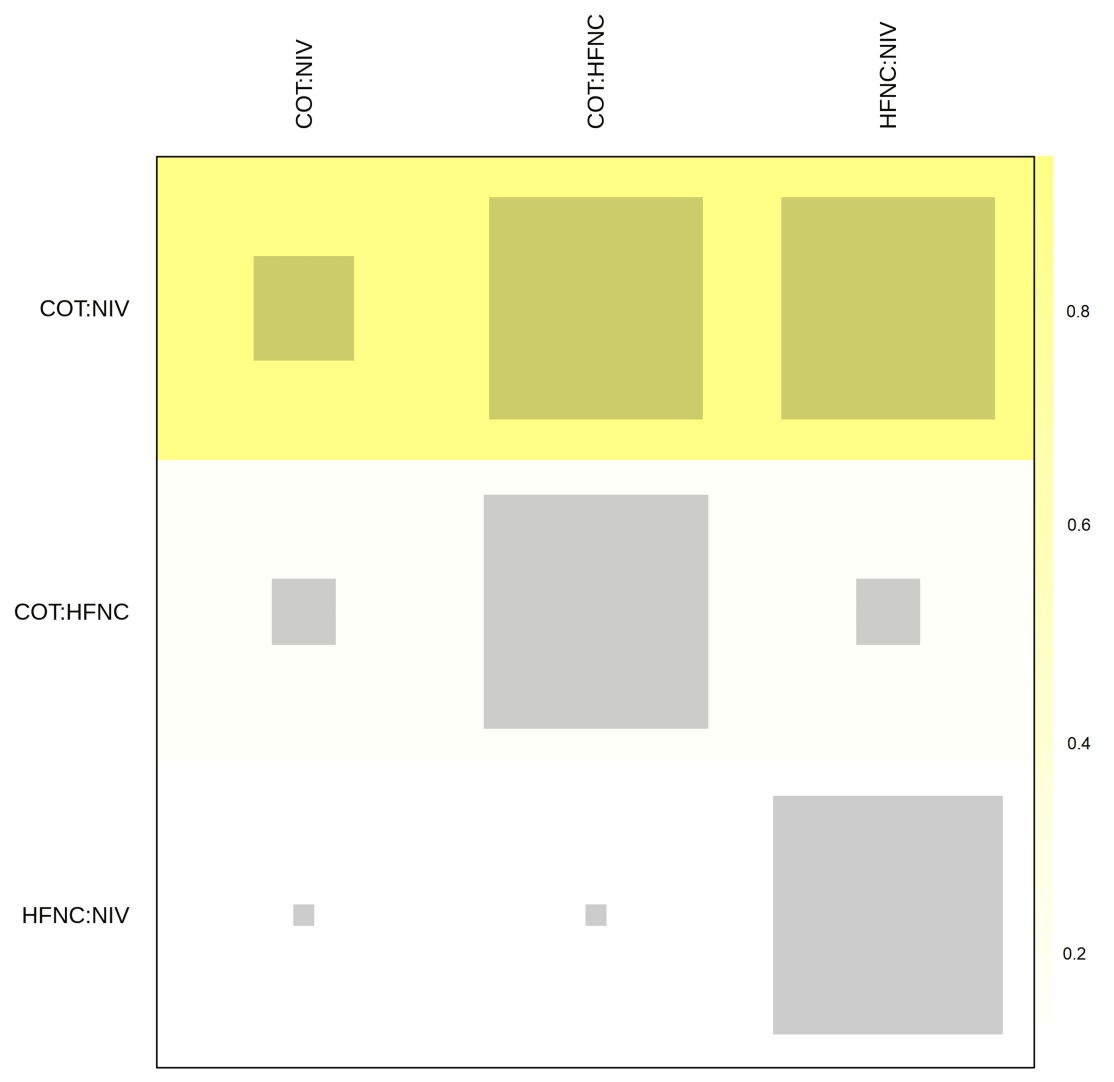
Net heat plot of the reintubation network Matrix visualization quantifying the contribution of direct comparisons to the network evidence and the extent of inconsistency. The size of the gray squares on the diagonal is proportional to the contribution of direct evidence from each design to the network estimate. The colored squares off the diagonal represent the inconsistency between direct and indirect evidence. Yellow squares indicate potential inconsistency, while the predominant gray squares observed here confirm the absence of major inconsistency within the network structure. Abbreviations: HFNC, High-Flow Nasal Cannula; NIV, Noninvasive Ventilation; COT, Conventional Oxygen Therapy.

Sensitivity analyses examining potential sources of this heterogeneity suggested that the inclusion of mixed populations (medical vs. surgical) contributed to this variance. Trials focusing exclusively on post-cardiac surgical patients [34,37] showed smaller effect sizes for HFNC compared to trials involving high-risk medical or abdominal surgery patients [26,28], likely due to the rapid reversibility of atelectasis in the former group. The consistency of the network structure was further verified visually using a net heat plot (Figure 6).

Secondary Outcome: PaO2/FiO2 Ratio

**Figure 7:**
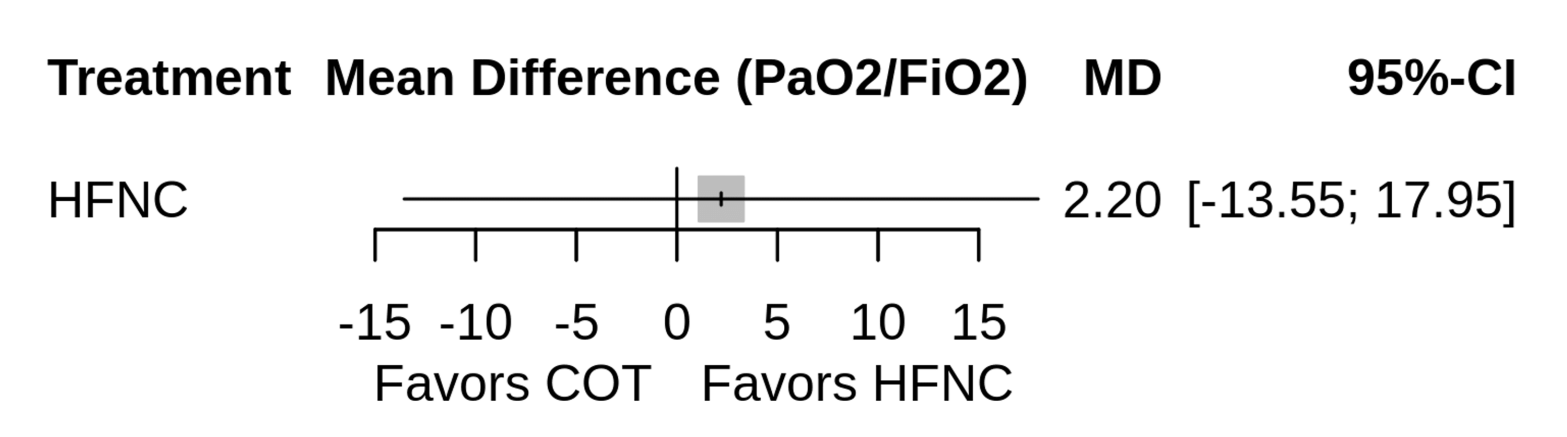
Forest plot of network meta-analysis results for the PaO2/FiO2 ratio Forest plot displaying the mean difference (MD) and 95% confidence intervals (CI) for the ratio of arterial oxygen partial pressure to fractional inspired oxygen (PaO2/FiO2) at 24 hours post-extubation. Results are based on a random-effects model compared against COT. Crossing the vertical line at 0 implies no significant difference.

Data regarding the PaO2/FiO2 ratio at 24 h were available for four studies comparing HFNC and COT [26,29,34,35]. The network meta-analysis showed no statistically significant difference in oxygenation between HFNC and COT (mean difference 2.20 mmHg; 95% CI -13.55 to 17.95), suggesting that while HFNC may reduce the work of breathing or reintubation, it does not necessarily result in higher arterial oxygenation ratios at the 24-hour mark in stabilized patients (Figure 7).

Publication Bias

**Figure 8:**
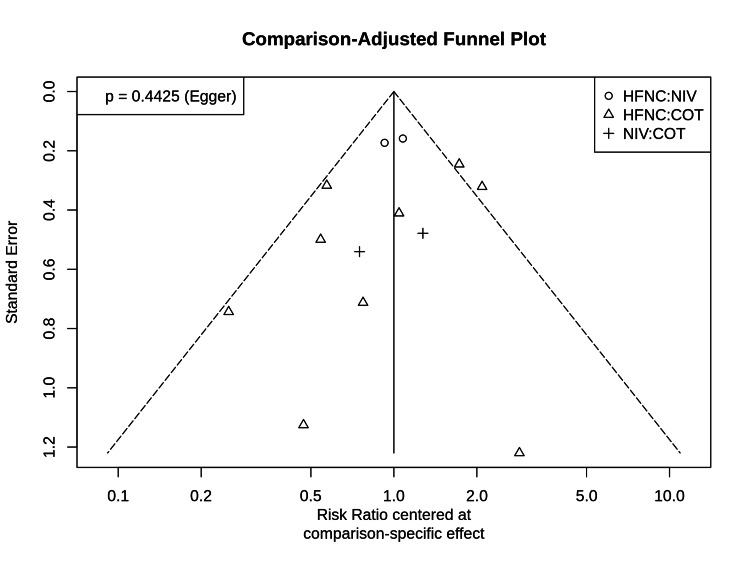
Comparison-adjusted funnel plot Funnel plot for the assessment of small-study effects and publication bias in the network. The vertical line represents the null effect, and the dashed lines represent the 95% confidence limits. Symmetry suggests an absence of significant publication bias (Egger’s test p = 0.44).

The comparison-adjusted funnel plot for reintubation (Figure 8) displays approximate symmetry, and Egger’s test did not indicate significant small-study effects or publication bias (p = 0.44).

Trial Sequential Analysis

**Figure 9:**
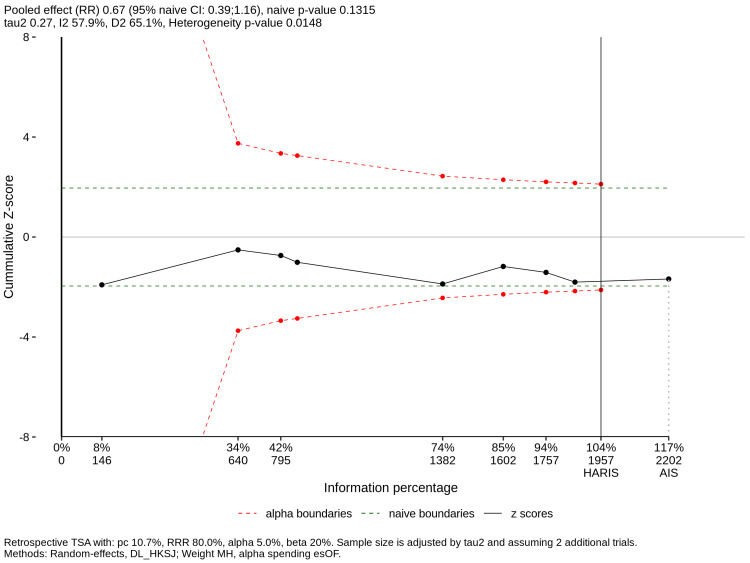
TSA for HFNC vs. COT; TSA for the outcome of reintubation in direct comparison studies The solid black line represents the cumulative Z-score. The horizontal red dashed lines represent the conventional significance boundaries (α = 0.05). The curved red dashed lines represent the trial sequential monitoring boundaries. The vertical line represents the required information size (RIS). Abbreviations: HFNC, High-Flow Nasal Cannula; COT, Conventional Oxygen Therapy; TSA, Trial Sequential Analysis

TSA was performed on the direct comparison between HFNC and COT (Figure 9). The cumulative Z-curve crossed the trial sequential monitoring boundary for benefit, and the required information size (RIS) of 1,957 patients was surpassed. Although the penalized confidence interval in the TSA crossed unity, aligning with the direct pairwise estimate (RR 0.71; 95% CI 0.48-1.04), this underscores the importance of the NMA. The NMA gained statistical precision by integrating indirect evidence from NIV trials, demonstrating a significant benefit (RR 0.68; 95% CI 0.47-0.97).

Certainty of Evidence (GRADE Assessment)

The certainty of the evidence was evaluated using the CINeMA framework (Table 2). The evidence supporting HFNC over COT for reducing reintubation was graded as moderate. We downgraded the risk of bias, as 100% of the included studies were unblinded owing to the nature of the intervention. We did not downgrade for inconsistency (node-splitting p > 0.05) or publication bias (funnel plot symmetry).

**Table 2:** GRADE evidence profile (CINeMA framework) for the outcome of reintubation [a] Contributing RCTs were open-label. Although outcome assessors were blinded in select studies, the treating physicians deciding on reintubation were aware of the intervention, introducing potential performance/detection bias. [b] The 95% confidence interval includes values that could support either intervention (clinically relevant benefit or harm), indicating imprecision. Source: Generated by the authors for this study using the CINeMA framework. Abbreviations: GRADE, Grading of Recommendations Assessment, Development, and Evaluation; CINeMA, Confidence in Network Meta-Analysis; RR, Risk Ratio; HFNC, High-Flow Nasal Cannula; COT, Conventional Oxygen Therapy; NIV, Noninvasive Ventilation.

Comparison	Network RR (95% CI)	Within-Study Bias	Reporting Bias	Incoherence	Imprecision	Heterogeneity	Certainty Level
HFNC vs. COT	0.68 (0.47 to 0.97)	Serious Concerns [a]	None	None	None	Some Concerns	MODERATE
NIV vs. COT	0.56 (0.34 to 0.94)	Serious Concerns [a]	None	None	None	None	MODERATE
HFNC vs. NIV	1.20 (0.76 to 1.89)	Serious Concerns [a]	None	None	Serious Concerns [b]	None	LOW

The evidence regarding the comparability of HFNC and NIV was graded as low. We downgraded for imprecision, as the 95% confidence interval included both appreciable benefit and harm (RR 1.20; 95% CI 0.76 to 1.89), and for risk of bias. The evidence supporting NIV over COT was graded as moderate, downgraded primarily for risk of bias in the direct evidence contributions.

Discussion

This NMA aggregates data from 14 RCTs to assess noninvasive strategies for respiratory support. The analysis confirms that HFNC provides a statistically significant advantage over standard oxygen therapy in averting reintubation. Moreover, the data suggest HFNC has comparable efficacy to NIV, supporting its role as a viable and often better-tolerated first-line option for post-extubation management across diverse critical care populations.

Interpretation of Primary Findings

The primary analysis indicates a 32% relative risk reduction in reintubation for patients treated with HFNC compared to COT (RR 0.68; 95% CI 0.47-0.97). This finding reinforces and extends previous meta-analyses [1]. By utilizing a network approach that incorporates indirect evidence from NIV trials, we improved the precision of the effect estimates. Physiologically, HFNC creates a PEEP effect that helps counteract the loss of functional residual capacity often observed after removing an endotracheal tube [6]. Furthermore, efficient washout of dead space enhances ventilation, thereby reducing the work of breathing during the transition to spontaneous respiration [7].

The analysis revealed no statistically significant difference in reintubation rates between the HFNC and NIV groups (RR 1.20; 95% CI, 0.76-1.89). This suggests that for the general post-extubation population, the theoretically superior pressure support provided by NIV does not translate into a statistically detectable reduction in reintubation compared to that provided by HFNC. This finding is clinically pivotal, as HFNC offers distinct practical advantages over NIV, including greater patient comfort, the ability to eat and speak, and a reduced risk of interface-related complications, such as skin breakdown [7,8]. HFNC may be the preferred modality for patients without a specific, absolute indication for NIV (e.g., hypercapnic respiratory failure).

Secondary Outcomes and Pathophysiological Insights

Regarding oxygenation, our analysis of the PaO2/FiO2 ratio at 24 hours showed no significant difference between the HFNC and COT groups. This finding contrasts with that of some individual trials [27,35] that reported superior oxygenation with HFNC. This discrepancy arises from the heterogeneity of the patient populations; our analysis included both hypoxemic medical and post-surgical patients. In post-cardiac surgery cohorts, atelectasis is often rapidly reversible, potentially diluting the observed benefit of HFNC on oxygenation parameters at the 24-hour mark [34,37]. It is also possible that while HFNC improves ventilatory efficiency (reduced respiratory rate and work of breathing), these benefits do not always manifest as a stark increase in arterial oxygen tension in stable patients already receiving titrated oxygen therapy.

Clinical Implications and Hierarchy of Treatment

SUCRA ranking analysis positioned NIV as the most effective intervention (88% probability), followed by HFNC (60%), with COT being the least effective (2%). While NIV ranks highest mathematically, the overlapping confidence intervals and the lack of statistical superiority over HFNC in the direct comparison suggest that the choice between HFNC and NIV should be nuanced. For patients with varying risk profiles, a stepped approach may be optimal: NIV remains the gold standard for patients with established hypercapnia or a high risk of extubation failure [4,39], whereas HFNC is an evidence-based, superior alternative to COT for the broader population of extubated patients [30]. The use of COT should be reserved for low-risk patients with minimal oxygen requirements, given its clearly inferior performance in preventing reintubation.

Strengths

The strengths of this study include the rigorous network methodology, which allowed for the first simultaneous comparison of all three major respiratory support modalities. TSA was utilized to confirm that the accumulated evidence for the HFNC vs. COT comparison was sufficient and robust, minimizing the risk of the Type I error associated with repetitive testing [23]. Furthermore, the inclusion of recent large-scale trials [26,27] ensured that our findings reflect current clinical practice.

Limitations

The certainty of evidence for the HFNC vs. NIV comparison was graded as low due to imprecision. This indicates that although the current data suggest equivalence, future large-scale trials could potentially demonstrate a difference. In addition, there was moderate statistical heterogeneity (I2≈48%) within the network, which is attributable to the clinical diversity of the included studies, which ranged from planned extubation in surgical patients [32,33] to rescue therapy in medical patients with acute respiratory failure [29]. Although a random-effects model was employed to account for this, the average treatment effect may not apply equally to all subpopulations. As with all respiratory support trials, blinding of the intervention was impossible, resulting in a high risk of performance bias across all included studies.

## Conclusions

This NMA demonstrated that HFNC is superior to COT in preventing reintubation in critically ill adults. HFNC shows comparable efficacy to NIV, supporting its use as a primary prophylactic strategy post-extubation. Clinicians should prioritize HFNC over COT to improve patient outcomes while reserving NIV for specific high-risk indications where pressure support is physiologically mandated. Future research should focus on identifying specific patient phenotypes (e.g., based on BMI or type of surgery) that might derive differential benefits from HFNC versus NIV to enable precision respiratory support.
